# Inactivation of Metabolic Genes Causes Short- and Long-Range dys-Regulation in *Escherichia coli* Metabolic Network

**DOI:** 10.1371/journal.pone.0078360

**Published:** 2013-12-05

**Authors:** Dinesh Kumar Barupal, Sang Jun Lee, Edward D. Karoly, Sankar Adhya

**Affiliations:** 1 Genome Center, University of California Davis, Davis, California, United States of America; 2 Infection and Immunity Research Center, KRIBB and University of Science and Technology, Daejeon, Korea; 3 Laboratory of Molecular Biology, Center for Cancer Research, National Cancer Institute, National Institutes of Health, Bethesda, Maryland, United States of America; 4 Metabolon, Inc., Durham, North Carolina, United States of America; University of Hyderabad, India

## Abstract

The metabolic network in *E. coli* can be severely affected by the inactivation of metabolic genes that are required to catabolize a nutrient (D-galactose). We hypothesized that the resulting accumulation of small molecules can yield local as well as systemic effects on the metabolic network. Analysis of metabolomics data in wild-type and D-galactose non-utilizing mutants, *galT, galU and galE*, reveal the large metabolic differences between the wild-type and the mutants when the strains were grown in D-galactose. Network mapping suggested that the enzymatic defects affected the metabolic modules located both at short- and long-ranges from the D-galactose metabolic module. These modules suggested alterations in glutathione, energy, nucleotide and lipid metabolism and disturbed carbon to nitrogen ratio in mutant strains. The altered modules are required for normal cell growth for the wild-type strain, explaining why the cell growth is inhibited in the mutants in the presence of D-galactose. Identification of these distance-based dys-regulations would enhance the systems level understanding of metabolic networks of microorganisms having importance in biomedical and biotechnological research.

## Introduction

The metabolic network of *E. coli* has been under active research of system biology using genomics, transcriptomics, proteomics and metabolomics. Genome-scale reconstruction of *E. coli* metabolism suggests 1366 genes, 2251 reactions and 1136 metabolites in the metabolic network of *E. coli* [1]. The network can be decomposed into multiple metabolic modules that may reflect several well known metabolic pathways such as TCA cycle, glycolysis or pyrimidine biosynthesis [2]. These modules have distinct cellular functions and provide building blocks for cell growth, cellular energy, reductive equivalents and signaling molecules. In bacteria under different nutrient regimes, different network modules are adjusted for adaption in the changed environment [3]. The metabolic network is tightly integrated with other molecular networks so a quick global response to altered conditions can be delivered [4,5]. For adaptation, several gene-regulatory mechanisms ensure the metabolic reprogramming that yield optimal qualitative and quantitative properties of different metabolic modules [6,7]. Elucidation of mechanistic changes in the metabolic network of an organism under genetic and environmental stress will expand our systems level understanding of metabolism and physiology. 

Systems level properties of metabolic networks, such as organization, robustness, topology, evolvability and global flux balancing are being actively studied in the wild-type. as well as in mutant strains of *E. coli* [8,9]. Flux-balance analysis of this network can predict metabolic flux distributions, growth rates and metabolic transport rates in *E. coli* [10]. Although these properties provide an abstract and global view of the metabolic network, the study of module level alterations is required to discover the biochemical mechanisms that are perturbed under genetical and environmental stresses. Addition of D-galactose to D-galactose non-utilizing mutants grown in another carbon source; for example, fructose causes cellular stress [11] leading to retarded cell growth. Since the metabolic network is highly connected [12,13], knocking down important metabolic genes can cause local, as well as systemic alterations in the metabolic network, and these alterations can be responsible for retarded cell growth. To test this hypothesis, wild-type and *galE*, *galT* and *galU* mutant strains of *E. coli* grown in galactose containing media were analyzed by a non-targeted metabolomics using mass spectrometry. The data were mapped into a metabolic network using the MetaMapp biochemical mapping approach [14]. We report that the inactivation of metabolic genes of D-galactose metabolism caused short- and long-range metabolic dys-regulations in the *E. coli* metabolic network. The altered modules are required for normal cell growth for the wild-type strain, explaining why the cell growth is inhibited in the mutants in the presence of D-galactose. 

## Results

**Figure 1:**
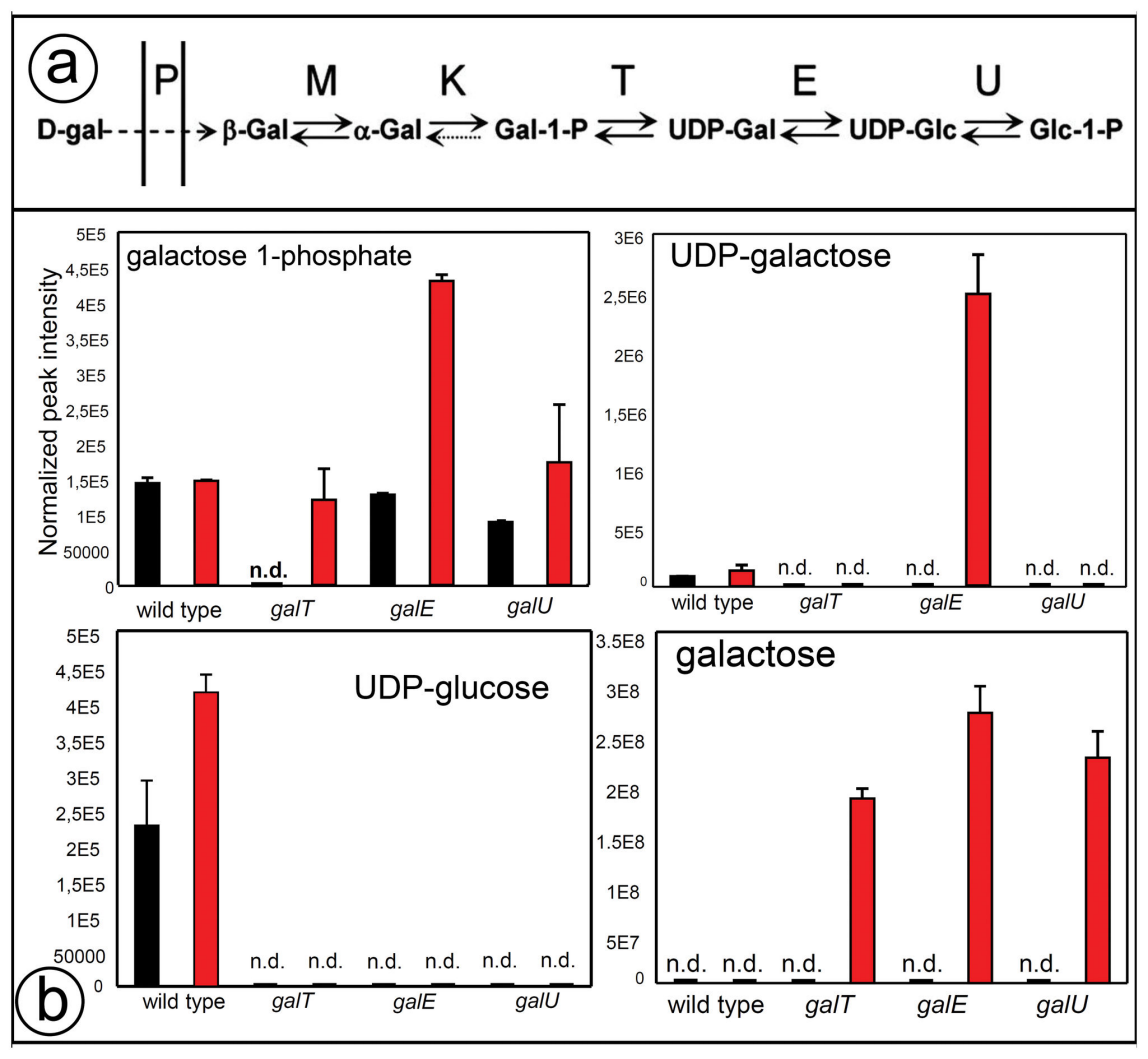
Inactivation of galactose metabolism pathways in *E. coli* mutants leads to disturbed levels of intermediates. (a) Upper panel shows the Leloir pathway in *E. coli* for the metabolism of galactose. (b) Lower panel shows the levels of intermediates in the pathways in wild-type and *galT, galE* and *galU* mutants. n=3 (biological replicates).

The biochemical pathway of D-galactose utilization in *E. coli* and the constituent enzymes are shown in Figure 1a. Cell growth of *E. coli* mutants, *galT, galU and galE*, encoding the enzymes Galactose-1-phosphate uridylyltransferase, UDPG synthatase, and Epimerase, respectively, in D-fructose minimal media are retarded in the presence of D-galactose, compared to the wild-type control [11]. Three replicates of wild-type and each mutant strain grown with or without D-galactose were collected for metabolomics analysis. A total of 157 metabolites were detected in *E. coli* using the combined approach of GCMS and LCMS (see Materials and Methods for details). The detected compounds cover a range of metabolic pathways including energy, nucleotide, lipids and amino acid metabolism (Table S1). The metabolomic survey confirmed metabolic consequences of inactivation of enzymes of D-galactose metabolism in *E. coli* (Figure 1b). D-galactose was not detected in the wild-type strain grown in the presence of sugar, suggesting a complete utilization of D-galactose into glucose-6-phosphate whereas D-galactose was accumulated in all three mutants. As expected, UDP-galactose was accumulated only in the *galE* mutants. The magnitude of galactose-1-phosphate accumulation in the *galE* mutant was higher in comparison to that in the other two mutants. UDP-glucose and glucose-1-phosphate were not detected in the mutant strains. Following validation of the expected metabolic consequences, we investigated the metabolomic dataset to identify the impact of the gene inactivation on the global metabolic variability and individual metabolic modules. 

To assess the global metabolic variability among all the strains, we calculated an unsupervised principal component analysis (PCA) model [15], which summarized the entire variability among metabolites into a limited number of vectors, known as principal components. Figure S1 shows the scatter plot of the first two principal components, which represent the maximum variance among samples. We observed that strains can be discriminated based on their metabolome when grown in D-galactose. Interestingly, the metabolic spectrum of the *galE* mutant without D-galactose was close to that of the wild-type strain. The large distances (overall variability) between mutants demonstrate profound metabolic alterations, in addition to the disturbance in the D-galactose metabolism.

**Figure 2:**
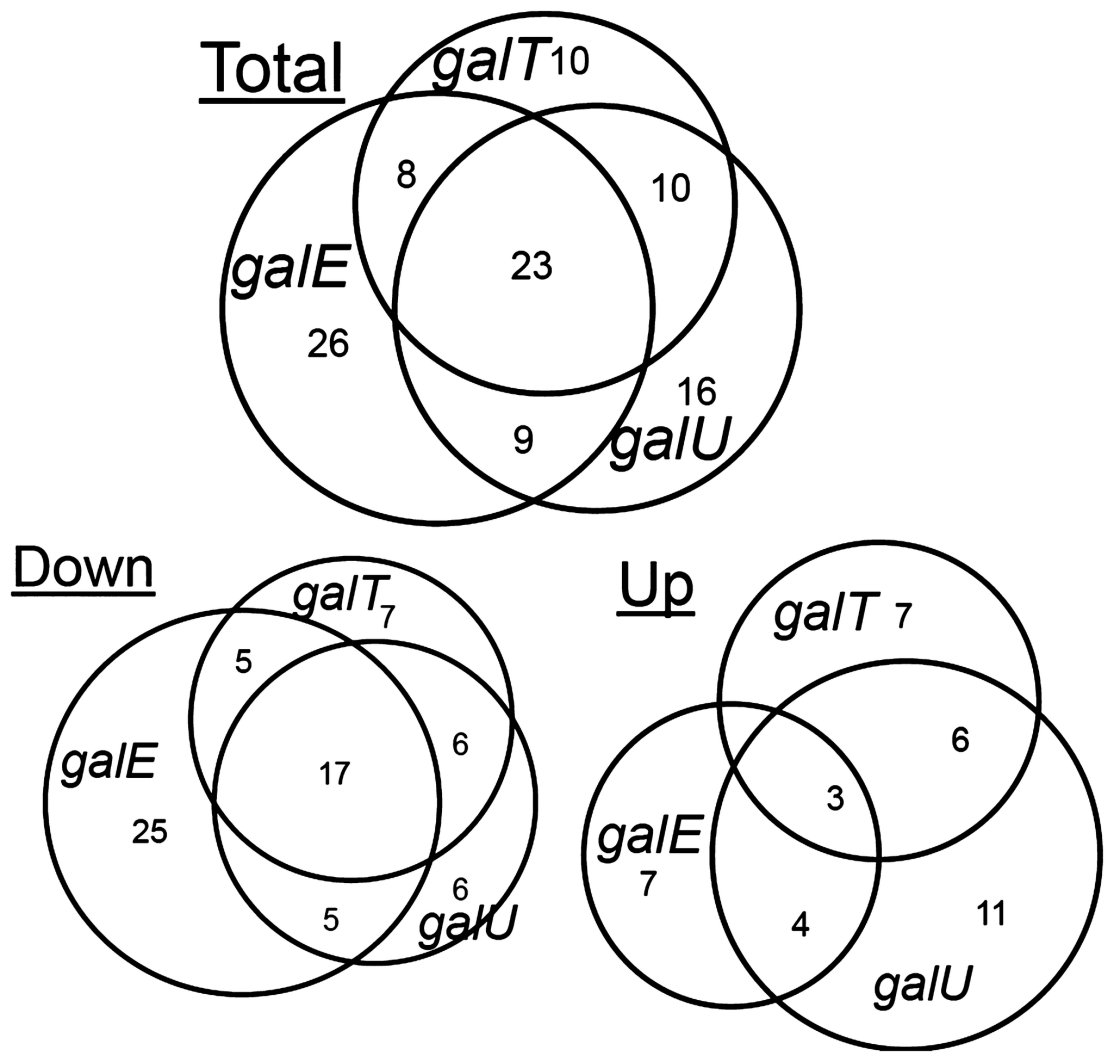
Significantly altered metabolites in different *E. coli* strains grown in galactose supplemented media. Top venn-diagrams show all the metabolites that were altered by galactose supplementation to three mutant strains. Numbers in parenthesis are total metabolites significantly affected by *gal* mutations. Bottom two venn-diagrams show the separated significantly increased and decreased metabolites following the galactose supplementation. Welch’s two sample T-test were calculated to identify the significantly altered metabolites at *p*-value less than 0.05 (n=3).

Whereas PCA provided a global view of metabolic variability among samples, the identification of significantly altered metabolites revealed the metabolites in different metabolic modules that were affected following the accumulation of D-galactose metabolic intermediates, which created cellular stress from within [11]. Welch’s two sample T-tests between strains grown with and without D-galactose addition were performed to identify the differentially altered metabolites on a p-value cutoff of <0.05. Only 22 metabolites were found to be altered in the wild-type strain (Table S2). Figure 2 shows the venn-diagrams to highlight the number of altered metabolites in different mutants. Up to 50 metabolites were significantly altered in each mutant strain (Tables S3-S5). A large number of compounds were down-regulated compared to the number of up-regulated metabolites in all the mutant strains, indicating the impeded metabolic pathways. The highest number of specifically down-regulated metabolites was found in the *galE* mutant, suggesting that metabolic response in this mutant was different from the other two strains, as observed in the PCA model. Up to 60% of the compounds that were decreased in the *galT* mutant were also found to be altered in the *galU* mutant, pointing to the common metabolic pathways that were affected by *galU* and *galT* mutations. A total of 23 metabolites were found to be altered in all three mutants, pointing toward common metabolic pathways disturbed in different mutants. Detailed results of ANOVA analysis is provided in Tables S2-S5.

**Figure 3:**
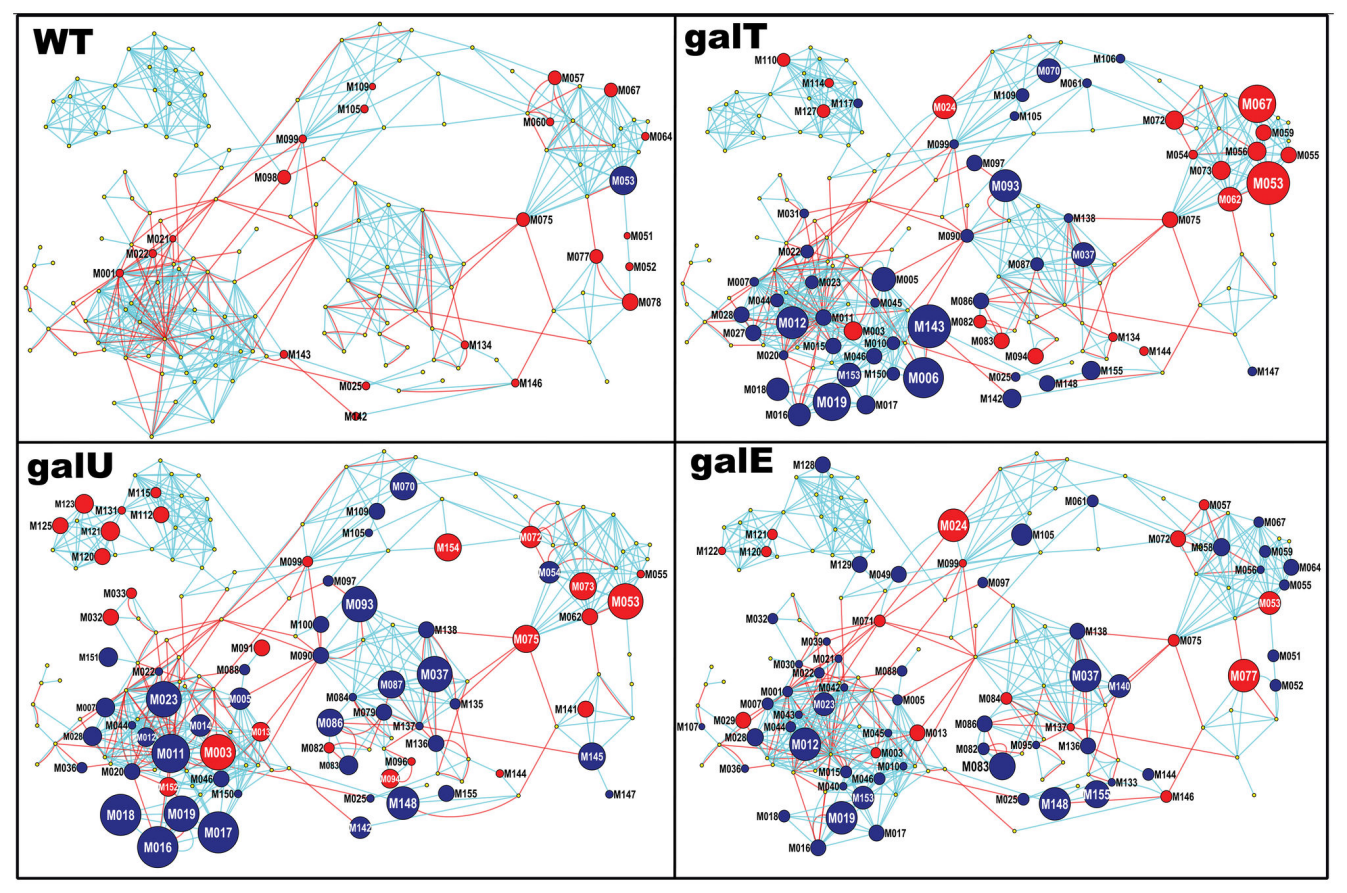
MetaMapp of the metabolic modules that were altered in *E. coli* grown in galactose. Each node is a metabolite, blue edges are chemical similarity links and red edges are KEGG RPAIR links as calculated by the MetaMapp software. Red nodes represent significantly increased metabolites, and blue nodes represent significantly decreased metabolites. Node sizes reflect the magnitude of fold change as provided in Tables S2-S5. Cytoscape organic layout algorithm was used to visualize the network graph, and results of Welch’s two sample T-test were overlaid on the graph. Labels of different modules are provided in the graph of wild-type strains. To maintain the clarity of the graph, metabolite labels were provided in Tables S2-S5 and in the graph metabolites are referenced with a number. Labels of big nodes are as follows: M053:galactose, M0067:glucose, M077:UDP-galactose, M073:glc/frc-1,6-bisphosphate, M075:ribose, M037:MTA, M093, Succinyl CoA, M143:dihydrooratate, M006:Ncarbomylphosphate, M019:GSSG, M003:asparagine, M011:glutamate, M018:cysteine-glutathione disulfide, M016:GSH, M017:opthalmate, M011:GABA, M023:homoserine, M148:5,6 dihydrouracil, M083:nicotinate ribonucleoside, and M140:guanosine.

Direct biological interpretation of the list of altered metabolites was difficult because of the large number of metabolic changes. Therefore, organizing the metabolites into metabolic modules was chosen as an approach to assist the interpretation of the results of ANOVA analyses. To do so, metabolites were mapped into a metabolic network using a recently developed biochemical mapping approach, MetaMapp [14]. The approach provided a solution to two situations: 1) that not all the metabolites in *E. coli* were present in the metabolomics datasets, and 2) that several detected metabolites were not present in the reconstructed metabolic network of *E. coli*. Since the metabolomics survey covers a variety of metabolites, five major clusters of metabolites were visually observed in the MetaMapp graph, similar to the modules observed previously [14] (Figure 3). Since, the network graph was composed of a smaller number of nodes, and the modules were clearly visible by the organic layout algorithm in Cytoscape, use of additional computational approaches (readily available within Cytsocape) for the module detection was not considered on this step of analysis. The Cytoscape visualization of the differentially regulated metabolites on this network graph showed that several metabolic modules located on a short distance, as well as long distance from D-galactose intermediates were severely altered (Figure 3). The distances can be interpreted as the number of reaction steps and/or the measure of chemical similarity between the two metabolites. Two closely located metabolites reflect that they are either highly similar to each other or they have very few biochemical reaction steps between them. The modular network graph enabled summarization of short- and long-range metabolic dys-regulations in the *E. coli* metabolic network, which are described in the next section. 

### Metabolite changes in gal mutants by D-galactose

In the presence of D-galactose, *galT, galU* and *galE* mutants showed biochemical alterations (Figure 3) that can be associated with retarded cell growth. The most striking finding was the inhibition of glutathione biosynthesis in all three mutants. Levels of GSH (M016), GSSG (M019), cysteine-glutathione S2 (M018) were many folds lower in the mutants. Levels of glutamate (M011) and glycine (M020), the key substrates of glutathione biosynthesis, were reduced in *galT* and *galU* mutants. Similarly, opthamalate (M017), which is an indicator of glutathione biosynthesis, was also decreased. Glutathione is a major anti-oxidant agent in cell [16], and its low level provides the first mechanism by which defects in D-galactose metabolism affects cell growth of *E. coli*. 

We identified an UTP-mediated relationship between pyrimidine biosynthesis and D-galactose metabolism. Higher pools of UDP-glucose were consumed to metabolize the higher amount of galactose that was imported in mutant strains. Increased biosynthesis of UTP from ribose, ATP and uracil is required to elevate the levels of UDP-glucose. For this reason, levels of uracil were maintained by reducing the catabolism of uracil as indicated by lower levels of dihydrouracil (M148) in *galU* and galE mutants. Increase in the levels of ribose (M075) in the mutants augments the UTP biosynthesis. Increase in ribose in all three mutants is likely caused by activation of riobnucleosidases in salvage pathways of purine and pyrimidines, because we have observed low levels of nucleosides including inosine (M135), adenosine (M138), guanosine (M140) and adenosine 5’ diphosphoribose (M087). The salvage of ribose from nucleosides resulted into a decrease in the levels of methylthioadenosine (MTA), which is an essential intermediate in methionine salvage and sperminine biosynthesis. Accumulation of ribose was highest in *galU* mutants as UTPs were not being utilized in this strain. However, in the *galT* mutant, UTPs are not utilized as shown [11] for the production of UDP-galactose. This signals the cells to lower the biosynthesis of uracil in *galT* mutants, leading to a large decrease in the levels of orotate (M142), dihydroorotate (143) and N-carbamylaspartate (M006), the key intermediates of pyrimidine biosynthesis. The salvage of substrates for biosynthesis is an important metabolic mechanism in adaptation to changing nutrients [17]. Since, synthesis from scratch would cost a large amount of energy; a transient salvage pathway would be more efficient mechanism to reprogram the metabolic network for galactose metabolism. However, in the mutants, low levels of nucleosides hampered the cell growth by disturbing DNA metabolism. 

Accumulation of D-galactose metabolism intermediates imbalanced the C/N ratio, which was sensed by the mutants as a low nitrogen condition in the presence of galactose. Low levels of glutamine/glutamate are sensed as a low nitrogen condition that induced nitrogen response genes [11]. As a result, consumption of key nitrogen compounds such as gamma-aminobutyric acid (GABA) (M012), homoserine (M023) and diaminopimelate (M028) was increased in mutants (Figure 3). Reduced homoserine is observed in *E. coli* under nutrient stress [18]. Levels of several amino acids were similar in control and galactose treated mutant strains, suggesting that *E. coli* tries to maintain the amino acids levels at the cost of some other nitrogen compounds. However, GABA is also an osmotic stress protectant and diaminopimelate is required for proper cell wall biosynthesis, indicating mechanisms that can inhibit the cell growth of the mutants. Recently it was discovered that GABA can also enter the TCA cycle as an energy source in nutrient starvation conditions [19]. Additionally, low levels of homoserine and MTA (M037) can be associated with decreased biosynthesis of homoserine lactone (HSL) [20]. This indicates that defects in galactose metabolism can negatively affect quorum sensing in *E. coli*. Accumulation of asparagine is a marker of protein degradation [21], indicating that mutants are utilizing proteins as a temporary nutrient source. The accumulation was highest in the *galU* mutant, suggesting that this mutant was under severe stress conditions, which is also reflected by the large fold changed in other metabolite levels (Figure 3). Inactivation of galactose metabolism genes created the environment that is sensed as a nutrient starving condition in *E. coli*, leading to activation of mechanisms such as consumption of GABA that sustain the functionality of the most essential metabolic pathways. 

Accumulation of galactose-1-phosphate in *galT* mutant attenuated the glucose phosphorylation. A large amount of ATP would be consumed to phosphorylate the imported galactose. As long as galactose-1- phosphate enters into glycolysis via glucose-6-phosphate, the ATP pool can be replenished. However, in the *galT* mutant, the phosphorylated galactose-1-phosphate (M062) is not metabolized further, and the ATP/ADP ration will be imbalanced. This will transiently affect the other ATP-dependent reactions 1 of them is glucokinase, which phosphorylate glucose is the first step to glycolysis. Consequently, accumulation of glucose was observed in *galT* mutant. Strikingly, the accumulated unphosphorylated glucose was converted into maltose (M055), maltotriose (M059) and mannose (M056) in *galT* mutants. We have increased levels of these sugars, as has been shown previously [22]. However, in *galE* mutants the levels of these sugars were lower compared to the controls, indicating the activation of maltose system by the accumulation of UDP-galactose and galactose. Levels of glucose and the diasacharides were unaffected in the *galU* mutant. *E. coli* mutants have imported the fructose in the media and converted it into fructose-6-phosphate (M072), which was converted into fructose 1,6, biphosphate (M073). Both compounds were increased in *galE* and *galT* mutants. Interestingly, *galU* consumed more fructose (M054) and low levels of fructose were observed in this mutant. Downstream metabolite in glycolysis, 3PGA (M070) was reduced in *galT* and *galU* mutants, indicating that glycolytic flux was rerouted toward pentose-phosphate pathway for the synthesis of ribose sugar (M075), which was found to be alleviated in all the mutants. 3-PGA is a precursor for synthesis of serine, glycine and the glycerol moiety in membrane lipids. Levels of serine were unaffected and lipids were increased in *galU* and *galT* mutants, suggesting the utilization of 3-PGA for anabolic reactions. In the *galU* mutant (Figure 3), low levels of acetyl CoA (M090) and citrate (M100) indicates the inefficient anaplerosis of the TCA cycle [23] in *galU* mutant from glycolysis and additional substrates. Distinctly, in the *galU* mutant (Figure 3), accumulation of lipids species, 2-oleoyl-GPE (M120), 1-palmitoyl-GPE (M121), 2-oleoyl-GPC (M125), 1-oleoyl-GPC (M123), 1-oleolglycerol (M131), myristoleate (M115) and palmitoleate (M112) was observed. The increase in lipids indicates that *galU* mutants are using lipids as an alternative source of energy, which is a metabolic response during the stationary phase. It has been reported that UDP-glucose is an important signaling molecule for the expression of genes related to the stationary phase [24]. A proper cell growth would need functional glycolysis and TCA cycle in bacterial cells, however trapping of phosphate by the imported galactose disturbed the energy metabolism in mutant strains, causing retarded cell growth of these mutants. 

### Metabolite changes in wild-type by D-galactose

While the metabolic network in mutants were severely affected by the inactivation of *gal* operon, the network in the wild-type strain showed adjustments that were needed to metabolize the galactose. For proper and rapid utilization of D-galactose, wild-type *E. coli* maintained high pools of intermediates of D-galactose, energy and pyrimidine metabolism (Figure 3). Elevated levels of the malate (M099) and fumarate (M098) were observed in wild-type cells grown in the presence of D-galactose. Biochemically closer compounds to D-galactose, which were increased, are glucose (M067), UDP-galactose (M077), N-acetylglucosamine (M052) and UDP-glucose (M078). Interestingly, increase in the pentose sugar ribose (M075) was accompanied by an increase in nearby nucleobases uracil (M146) and hypoxanthine (M134). Increase in di-hydroorotate (M143) indicates activated pyrimidine biosynthesis for production of UTP. PRPP (5-phospho-α-D-ribose-1-diphosphate) is a common precursor for histidine and pyrimidine biosynthesis and D-galactose addition in media diverted more carbon flux in PRPP synthesis, which caused an increase in the levels of histidine (M025). Increased alanine (M002) and N-acetylglucusamine (M052) can be utilized for the synthesis of peptidoglycan monomers, which also depends on the UTP biosynthesis. Increase in sorbitol-6-phosphate (M060) and mannitol-6-phosphate (M057) suggests an increase in the expression of *mtlD* and *srlD* genes. It has been previously reported that mannitol-1-phosphate can be converted into ribose sugar [25], pointing towards a yet to be discovered relationship between *gal* operon and mannitol operon. 

## Discussion

When *E. coli* senses the presence of an alternative nutrient, it introduces the required changes in the metabolic network to metabolize the available nutrient [26]. In case of galactose, the gal operon is activated to produce necessary enzymes to catabolize galactose. In addition, the substrates for galactose metabolism such as UDP-glucose would be synthesized at a higher rate. We found that after successfully switching to galactose, the metabolic network in wild-type strain reaches a steady-state condition in which several metabolic pathways are activated to ensure the complete catabolism of galactose. Because metabolic network is highly connected [13], activation of one metabolic pathway can also affect nearby pathways, especially those having common precursors. However, in the *E. coli* mutants that cannot metabolize galactose, the initial investment in supporting metabolic pathways after sensing the extracellular galactose was not recovered, and it resulted in the accumulation of galactose metabolic intermediates [11], the energy stress and unbalancing of several other interconnected metabolic pathways. These pathways represent the short- and long-range dys-regulations caused by the inactivation of important metabolic genes in metabolic networks. We have concluded that retarded cell growth of *E. coli* is attributed to these impaired modules. Our study complements the global analyses of metabolic networks in bacteria [3,9] by detailing the module level changes that occur when an organism is under genetic and environmental stress. We have discovered an alteration of metabolic modules, such as glycolysis and maltose systems located at short biochemical distances from the D-galactose metabolism module, as well as of modules such as glutathione and pyrimidine metabolisms located at long distances from the D-galactose module in the metabolic network in *gal* mutants grown in the presence of D-galactose. Since the analytical methods in our study covers only a portion of *E. coli* metabolome, further advances in metabolomics are expected to identify more metabolites and detect low-abundant metabolites [27]. Availability of additional metabolite data can identify more precise changes in metabolic networks of the mutants of galactose metabolism. 

Discovery of short- and long-range metabolic dys-regulation and its effects on cellular growth can have significant impact on biomedical and biotechnological research. Identification of novel drug targets in bacteria is important [28] because of enhanced antibiotic resistance by persistent strains [29]. The mechanisms that we have identified in the present study can be explored in the direction of finding novel targets to stop the growth of harmful bacteria. Changes in the composition of gut microflora have been implicated in several diseases [30]. Nutrient availability within the colon can significantly influence the growth of a specific microflora that cannot survive in the altered niche. Integration of metagenomics and metabolomics datasets can associate different metabolic genes in metagenome to short- and long-range metabolic dys-regulations in gut-microflora in the human colon. Discovery of these associations can refine existing models for the roles of gut microflora in gastrointestinal disorders. Mutant strains of *E. coli* can be proposed for the large scale production of commercially important biomolecules [31,32]. Identification of the right type of mutant strain is always a prerequisite for industrial scale production of biomolecules. Metabolomics-driven identification of biochemical mechanisms, as presented in our study, can contribute greatly in ranking the mutants that can be further optimized by additional genetic engineering to meet the scaling-up requirements.

Chock-points in a metabolic network are the essential components (genes, enzymes and metabolites) for cellular growth, from which the highest number of shortest paths are passing through. Using graph theory algorithms, computational identification of choke-points have been undertaken in microorganisms [33], and they can be experimentally validated by knocking out the genes or blocking its function by a chemical. Choke-points are suggested as important drug targets to kill pathogenic microorganisms [34,35]. We have observed the growth retardation for all the three mutants, therefore these genes are experimentally validated choke-points in the metabolic network of *E. coli*. The importance of these three genes in cell growth has been observed several times, however the underlying molecular mechanisms that led to growth retardation are not yet discovered. Therefore, our analysis complements the computational/experimental identification of choke-points, by providing a further mechanistic view responsible for the growth retardation. Furthermore, the data can also be utilized to validate the computational prediction of metabolites as choke-points in the metabolic networks of *E. coli*.

In summary, inactivation of a single gene function can cause enormous metabolic changes outside the know biochemical neighborhood of the encoded enzymes consistent with our global transcription analysis that showed dys-regulation of genes near and far from the function of the inactivated genes. Natural selection eliminates the mutants that have failed to survive in a changed environment [36]. But the responsible mechanisms in a metabolic network that lead to the survival failure are understudied as evolutionary studies focus mostly on observation of the growth of the survived mutants [37]. Our study highlighted the precise biochemical alterations that can be responsible for elimination of mutants during the natural selection events involving nutrient pressure. Investigation of mutants of other metabolic pathways would help to build a compendium of short- and long-range metabolic dys-regulations. The compendium can refine our understanding of natural selection of *E. coli* mutants.

## Materials and Methods

### Bacteria, plasmids and bacteriophages

Bacteriophage P1 lysates of Keio collection of *E. coli* deletion mutants were used to transduce *galT* (UniProt ID : P07902)*, galE* (UniProt ID : Q14376) and *galU* (UniProt ID : P0AEP3) mutations carrying kanamycin markers into BW25113 wild-type strain to make isogenic mutant strains. Subsequently, the pCP20 [25] used for recombineering were transformed into the mutants to delete the kanamycin substitution marker in each case to avoid an unwanted transcriptional polarity in *gal* gene expression in the operon. After removing the kanamycin marker, the temperature-sensitive pCP20 plasmid was cured at 42°C. P1 transduction was also carried out to introduce *glpR* mutation into the *galT* mutant cells to make the *galT glpR* double mutant.

### Metabolomic analysis

Bacterial cells were grown in 125-mL corning flasks containing 30 mL of M63 minimal medium plus 0.3% fructose at 37°C. At OD_600_ of 0.6, the cultures were divided into two flasks. D-galactose (final 0.3%) was added to one and cells were further grown for 0.5h. Cells were then placed on ice and harvested. The pellet was gently washed and re-suspended with PBS solution. The cell pellet was collected and stored at -80°C. Each sample is accessioned into a LIMS system, assigned a unique identifier, and stored at -70°C. To remove protein, to dissociate small molecules bound to protein or trapped in the precipitated protein matrix, and to recover chemically diverse metabolites, proteins are precipitated with methanol by vigorous shaking for 2 minutes in a Glen Mills Genogrinder 2000. The samples were centrifuged, supernatant removed (MicroLab STAR® robotics), and split into equal volumes for analysis on the LC+, LC-, and GC platforms by Metabolon, Inc (NC, USA). One aliquot was retained for backup analysis, if needed. Raw data matrix is provided in Table S1. 

### Statistical analysis and MetaMapp mapping

Welch’s two sample T-tests were used to identify the significantly altered metabolites. R and Statistica data miner Version 10 were used for statistical analysis. Mole-file encoded chemical structures of the identified metabolites were retrieved from the PubChem database. A pair-wise Tanimoto chemical similarity matrix was calculated for the structures on the PubChem website. We downloaded the similarity matrix and used it as an input for MetaMapp software [14] available at www.metamapp.fiehnlab.ucdavis.edu. A single-step metabolic reaction network was calculated using KEGG RPAIR computations. Chemical similarity and KEGG RPAIR networks were merged into a single graph, which was visualized in Cytoscape. The Cytoscape Session file is provided in Figure S2. 

## Supporting Information

Figure S1 **Principal component analysis of metabolome profiles of wild-type, *galT, galU*, and *galE* strains in the presence or absence of D-galactose.**
(TIF)

Figure S2 **Cytoscape Session file (no title).**
(XML)

Table S1 **List of compounds detected by GCMS and LCHS in wild-type and mutant *E. coli* strains.**
(XLSX)

Table S2 **Significantly altered (Student’s T-test, *p*<0.05) metabolites in wild-type strain of *E. coli* cultured in galactose supplemented media.**
(DOCX)

Table S3 **Significantly altered (Student’s T-test, *p*<0.05) metabolites in *galT* mutant strain of *E. coli* cultured in galactose supplemented media.**
(DOCX)

Table S4 **Significantly altered (Student’s T-test, *p*<0.05) metabolites in *galE* mutant strain of *E. coli* cultured in galactose supplemented media.**
(DOCX)

Table S5 **Significantly altered (Student’s T-test, *p*<0.05) metabolites in *galU* mutant strain of *E. coli* cultured in galactose supplemented media.**
(DOCX)
