# Supplementary figures and images for: Inactivation of Metabolic Genes Causes Short- and Long-Range dys-Regulation in Escherichia coli Metabolic Network
Source: PLoS One. 2013 Dec 5;8(12):e78360. doi: 10.1371/journal.pone.0078360 (PMC3868466; doi:10.1371/journal.pone.0078360)

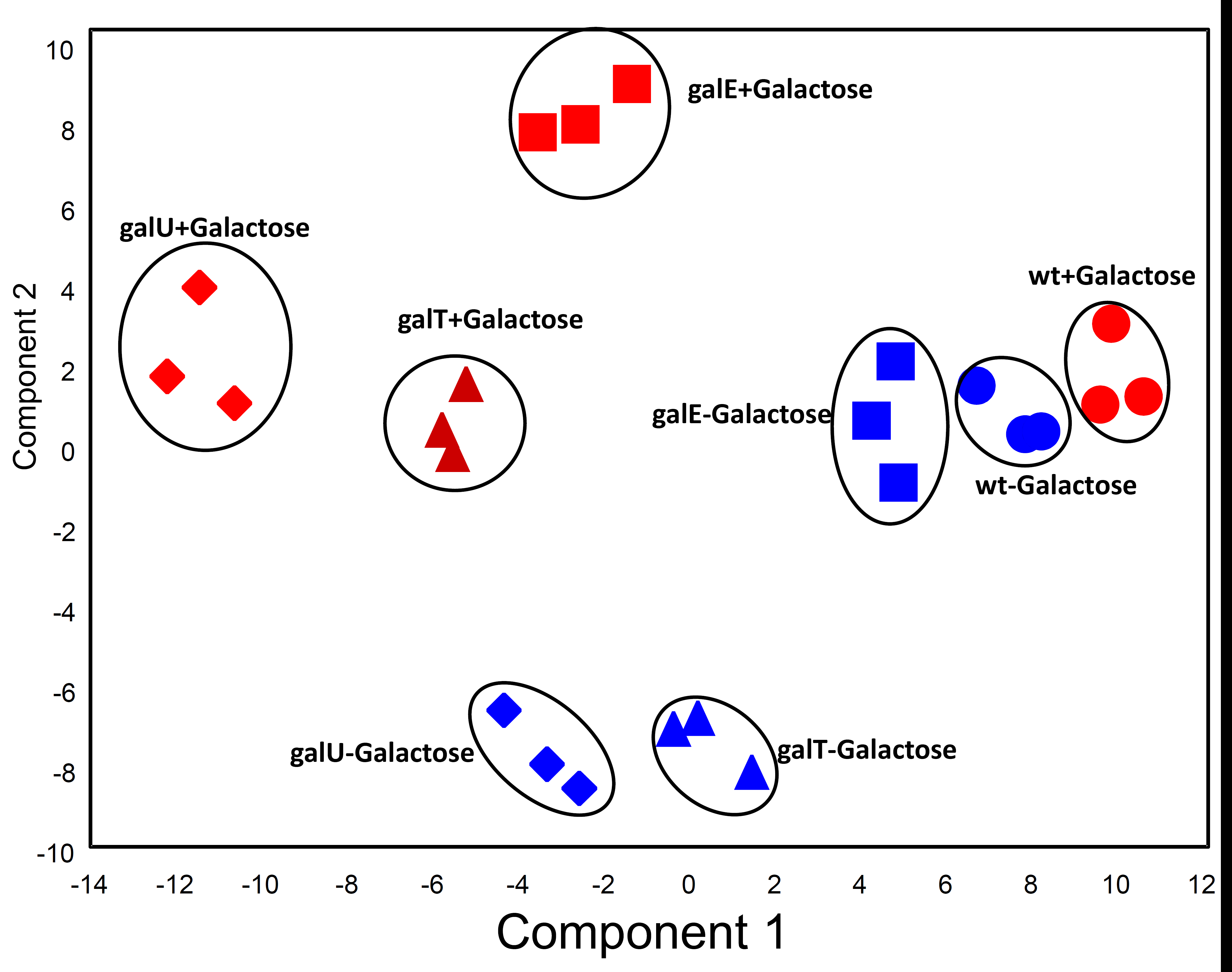

Supplement: Figure S1 — Principal component analysis of metabolome profiles of wild-type, galT, galU, and galE strains in the presence or absence of D-galactose. (TIF) [file pone.0078360.s001.tif]
