# Supplementary material for: Inactivation of Metabolic Genes Causes Short- and Long-Range dys-Regulation in Escherichia coli Metabolic Network
Source: PLoS One. 2013 Dec 5;8(12):e78360. doi: 10.1371/journal.pone.0078360 (PMC3868466; doi:10.1371/journal.pone.0078360)
Supplement: Table S2 — Significantly altered (Student’s T-test, p<0.05) metabolites in wild-type strain of E. coli cultured in galactose supplemented media. (DOCX) [file pone.0078360.s004.docx]

Table S2. Significantly altered (Student TTest, p<0.05) metabolites in wild-type *strain of E. Coli* cultured in galactose supplemented media.

| **Metabolite Name** | **PubChem ID** | **Label in Fig. 3** | **WT p-value** | **WT fold change** |
| --- | --- | --- | --- | --- |
| 4-hydroxybutyrate (GHB) | 10413 | M105 | 0.00010941 | 1.31 |
| galactose | 439357 | M053 | 0.000420931 | 0.30 |
| uracil | 1174 | M146 | 0.001945672 | 1.31 |
| orotate | 967 | M142 | 0.002804134 | 1.25 |
| hypoxanthine | 790 | M134 | 0.003385362 | 1.31 |
| ribose | 5779 | M075 | 0.004204769 | 1.58 |
| alanine | 5950 | M001 | 0.006709157 | 1.26 |
| UDP-glucose | 53477679 | M078 | 0.007563828 | 1.83 |
| sorbitol 6-phosphate | 21872877 | M060 | 0.009120471 | 1.36 |
| glucose | 5793 | M067 | 0.010583949 | 1.76 |
| 3-hydroxybutyrate (BHBA) | 441 | M109 | 0.012714272 | 1.12 |
| dihydroorotate | 648 | M143 | 0.019271154 | 1.47 |
| threonine | 6288 | M022 | 0.022616243 | 1.23 |
| glucosamine | 439213 | M051 | 0.025559 | 1.18 |
| mannitol-1-phosphate | 130418 | M057 | 0.027739065 | 1.72 |
| serine | 5951 | M021 | 0.034023659 | 1.09 |
| malate | 525 | M099 | 0.035423827 | 1.34 |
| histidine | 6274 | M025 | 0.037239595 | 1.31 |
| UDP-galactose | 46936243 | M077 | 0.039954473 | 1.54 |
| N-acetylglucosamine | 439174 | M052 | 0.040503326 | 1.44 |
| fumarate | 723 | M098 | 0.040819648 | 1.59 |
| maltotetraose | 439639 | M064 | 0.042729031 | 1.23 |
