# Supplementary material for: Inactivation of Metabolic Genes Causes Short- and Long-Range dys-Regulation in Escherichia coli Metabolic Network
Source: PLoS One. 2013 Dec 5;8(12):e78360. doi: 10.1371/journal.pone.0078360 (PMC3868466; doi:10.1371/journal.pone.0078360)
Supplement: Table S3 — Significantly altered (Student’s T-test, p<0.05) metabolites in galT mutant strain of E. coli cultured in galactose supplemented media. (DOCX) [file pone.0078360.s005.docx]

Table S3. Significantly altered (Student TTest, p<0.05) metabolites in *galT* strain of *E. Coli* cultured in galactose supplemented media.

| **Metabolite Name** | **PubChem** | **Label in Fig. 3** | **galT p-value** | **galT-fold change** |
| --- | --- | --- | --- | --- |
| N-acetylaspartate (NAA) | 65065 | M005 | 7.74E-07 | 0.23 |
| galactose | 439357 | M053 | 9.32E-06 | 1135.61 |
| pseudouridine | 15047 | M147 | 3.13E-05 | 0.73 |
| glucose | 5793 | M067 | 8.01E-05 | 14.55 |
| ophthalmate | 193304 | M017 | 0.000102 | 0.29 |
| ribose | 5779 | M075 | 0.00012 | 2.85 |
| orotate | 967 | M142 | 0.000201 | 0.28 |
| mannose | 18950 | M056 | 0.000249 | 3.74 |
| nicotinate adenine dinucleotide (NAAD+) | 165490 | M086 | 0.000296 | 0.46 |
| 3-hydroxybutyrate (BHBA) | 441 | M109 | 0.00039 | 0.63 |
| asparagine | 6267 | M003 | 0.000408 | 3.52 |
| riboflavin (Vitamin B2) | 6759 | M094 | 0.000474 | 2.67 |
| glutathione, oxidized (GSSG) | 975 | M019 | 0.000557 | 0.08 |
| 3-phosphoglycerate | 724 | M070 | 0.000664 | 0.20 |
| Isobar: fructose 1,6-diphosphate, glucose 1,6-diphosphate | 718 | M073 | 0.000733 | 3.29 |
| nicotinic acid mononucleotide (NaMN) | 121991 | M080 | 0.001018 | 1.53 |
| acetyl CoA | 444493 | M090 | 0.001158 | 0.53 |
| dihydroorotate | 648 | M143 | 0.001169 | 0.03 |
| maltose | 439186 | M055 | 0.001257 | 2.44 |
| 2-aminobutyrate | 80283 | M007 | 0.001531 | 0.71 |
| N-carbamoylaspartate | 93072 | M006 | 0.001941 | 0.04 |
| succinate | 1110 | M097 | 0.002446 | 0.49 |
| valine | 1182 | M044 | 0.002727 | 0.53 |
| hypoxanthine | 790 | M134 | 0.002894 | 1.14 |
| 10-nonadecenoate (19:1n9) | 5312513 | M114 | 0.002902 | 1.46 |
| 5,6-dihydrouracil | 649 | M148 | 0.002904 | 0.42 |
| homoserine | 12647 | M023 | 0.003426 | 0.54 |
| cysteine-glutathione disulfide | 3080690 | M018 | 0.003499 | 0.25 |
| N-acetylvaline | 227752 | M046 | 0.003892 | 0.49 |
| threonine | 6288 | M022 | 0.005133 | 0.66 |
| nicotinate | 938 | M082 | 0.005283 | 1.86 |
| glutathione, reduced (GSH) | 124886 | M016 | 0.005601 | 0.25 |
| diaminopimelate | 865 | M028 | 0.005699 | 0.38 |
| gamma-aminobutyrate (GABA) | 119 | M012 | 0.006238 | 0.12 |
| glycine | 750 | M020 | 0.008281 | 0.71 |
| histidine | 6274 | M025 | 0.009833 | 0.70 |
| alanylalanine | 601 | M150 | 0.010289 | 0.66 |
| thymine | 1135 | M144 | 0.010462 | 1.26 |
| adenosine 5'diphosphoribose | 192 | M087 | 0.012516 | 0.61 |
| glutamate | 23327 | M011 | 0.013469 | 0.47 |
| tyrosine | 6057 | M031 | 0.013683 | 0.76 |
| N-acetylmethionine | 6180 | M010 | 0.016288 | 0.62 |
| caprylate (8:0) | 379 | M127 | 0.016845 | 1.69 |
| glutamine | 5961 | M015 | 0.017628 | 0.45 |
| nicotinate ribonucleoside* | 121991 | M083 | 0.022956 | 2.38 |
| maltotriose | 439586 | M059 | 0.025537 | 2.94 |
| adenosine | 60961 | M138 | 0.025732 | 0.78 |
| lysine | 5962 | M027 | 0.027513 | 0.37 |
| glycerol 3-phosphate (G3P) | 439162 | M106 | 0.028911 | 0.75 |
| 2-pyrrolidinone | 12025 | M155 | 0.033861 | 0.32 |
| N-acetylisoleucine | 306109 | M045 | 0.035543 | 0.84 |
| cis-vaccenate (18:1n7) | 5282761 | M110 | 0.038427 | 1.98 |
| malate | 525 | M099 | 0.045267 | 0.83 |
| fructose | 439709 | M054 | 0.045853 | 1.15 |
| myristate (14:0) | 11005 | M117 | 0.047681 | 0.86 |
| 4-hydroxybutyrate (GHB) | 10413 | M105 | 0.048354 | 0.81 |
| tagatose | 92092 | M061 | 0.048426 | 0.67 |
| fructose-6-phosphate | 69507 | M072 | Significant | UP |
| 5-methylthioadenosine (MTA) | 149 | M037 | Significant | DOWN |
| galactose 1-phosphate | 123912 | M062 | Significant | UP |
| succinyl CoA | 439161 | M093 | Significant | Down |
| gamma-glutamylglutamate | 92865 | M153 | Significant | Down |
