# Supplementary material for: Inactivation of Metabolic Genes Causes Short- and Long-Range dys-Regulation in Escherichia coli Metabolic Network
Source: PLoS One. 2013 Dec 5;8(12):e78360. doi: 10.1371/journal.pone.0078360 (PMC3868466; doi:10.1371/journal.pone.0078360)
Supplement: Table S4 — Significantly altered (Student’s T-test, p<0.05) metabolites in galE mutant strain of E. coli cultured in galactose supplemented media. (DOCX) [file pone.0078360.s006.docx]

Table S4. Significantly altered (Student TTest, p<0.05) metabolites in *galE* strain of *E. Coli* cultured in galactose supplemented media.

| **Metabolite Name** | **PubChem ID** | **Labels in Fig. 3** | **galE p-value** | **galE fold change** |
| --- | --- | --- | --- | --- |
| gamma-aminobutyrate (GABA) | 119 | M012 | 1.09E-06 | 0.107229 |
| homoserine | 12647 | M023 | 2.3E-05 | 0.206173 |
| galactose | 439357 | M053 | 2.82E-05 | 4227.291 |
| glucosamine | 439213 | M051 | 7.63E-05 | 0.355439 |
| nicotinate adenine dinucleotide (NAAD+) | 165490 | M086 | 7.79E-05 | 0.312713 |
| 4-hydroxybutyrate (GHB) | 10413 | M105 | 9.15E-05 | 0.259606 |
| N-acetylaspartate (NAA) | 65065 | M005 | 9.4E-05 | 0.516967 |
| threonine | 6288 | M022 | 0.000142 | 0.547385 |
| thymine | 1135 | M144 | 0.000148 | 0.478397 |
| 2-aminobutyrate | 80283 | M007 | 0.000175 | 0.483039 |
| glutathione, reduced (GSH) | 124886 | M016 | 0.000333 | 0.294948 |
| ophthalmate | 193304 | M017 | 0.000437 | 0.294286 |
| ribose | 5779 | M075 | 0.000636 | 2.015833 |
| valine | 1182 | M044 | 0.000696 | 0.559744 |
| adenine | 190 | M136 | 0.00081 | 0.32701 |
| N-acetylglutamate | 185 | M013 | 0.000851 | 3.512047 |
| N-acetylmethionine | 6180 | M010 | 0.001137 | 0.721181 |
| mannitol | 6251 | M058 | 0.001269 | 0.27495 |
| pantothenate | 6613 | M088 | 0.001359 | 0.524653 |
| nicotinamide adenine dinucleotide reduced (NADH) | 439153 | M084 | 0.001993 | 2.608458 |
| adenosine | 60961 | M138 | 0.002119 | 0.317051 |
| uracil | 1174 | M146 | 0.0023 | 2.29539 |
| glutamine | 5961 | M015 | 0.002437 | 0.447924 |
| histidine | 6274 | M025 | 0.002527 | 0.503614 |
| proline | 145742 | M040 | 0.002962 | 0.678255 |
| maltose | 439186 | M055 | 0.003102 | 0.514439 |
| 2-pyrrolidinone | 12025 | M155 | 0.003306 | 0.148255 |
| phenylalanine | 6140 | M030 | 0.003466 | 0.725735 |
| diaminopimelate | 865 | M028 | 0.004552 | 0.266712 |
| pyruvate | 1060 | M071 | 0.004725 | 2.335769 |
| 2-oleoylglycerophosphoethanolamine* | 9547071 | M120 | 0.005242 | 1.733701 |
| N-acetylisoleucine | 306109 | M045 | 0.006369 | 0.719699 |
| mannose | 18950 | M056 | 0.00643 | 0.769822 |
| glutathione, oxidized (GSSG) | 975 | M019 | 0.006886 | 0.117654 |
| 1-palmitoylglycerophospho- ethanolamine | 89229 | M121 | 0.007145 | 1.70883 |
| nicotinate | 938 | M082 | 0.007263 | 0.502881 |
| succinate | 1110 | M097 | 0.007777 | 0.615762 |
| maltotetraose | 439639 | M064 | 0.009281 | 0.657476 |
| phenyllactate (PLA) | 643327 | M032 | 0.009749 | 0.631315 |
| glucose | 5793 | M067 | 0.010393 | 0.627247 |
| gamma-glutamylglutamate | 92865 | M153 | 0.010554 | 0.231833 |
| agmatine | 199 | M036 | 0.010639 | 0.680763 |
| N-acetylglucosamine | 439174 | M052 | 0.011633 | 0.416004 |
| alanine | 5950 | M001 | 0.012758 | 0.605882 |
| isoleucine | 791 | M043 | 0.01488 | 0.703075 |
| leucine | 6106 | M042 | 0.016484 | 0.741484 |
| serine | 5951 | M021 | 0.016607 | 0.750093 |
| fructose-6-phosphate | 69507 | M072 | 0.018278 | 3.3039 |
| Isobar: ribulose 5-phosphate, xylulose 5-phosphate | 0 | M076 | 0.019285 | 0.647061 |
| tryptophan | 6305 | M039 | 0.021067 | 0.753353 |
| xanthine | 1188 | M133 | 0.021451 | 0.681246 |
| maltotriose | 439586 | M059 | 0.021677 | 0.403888 |
| nicotinate ribonucleoside* | 121991 | M083 | 0.024705 | 0.126566 |
| tagatose | 92092 | M061 | 0.02718 | 0.474414 |
| flavin adenine dinucleotide (FAD) | 439154 | M095 | 0.028339 | 0.857559 |
| cysteine-glutathione disulfide | 3080690 | M018 | 0.03225 | 0.347367 |
| mannitol-1-phosphate | 130418 | M057 | 0.035282 | 1.879193 |
| adenosine 5'-monophosphate (AMP) | 6083 | M137 | 0.037819 | 1.411722 |
| 1-oleoylglycerophosphoethanolamine | 25244076 | M122 | 0.038554 | 1.363146 |
| ethanolamine | 700 | M107 | 0.038777 | 0.906218 |
| asparagine | 6267 | M003 | 0.040139 | 1.665463 |
| malate | 525 | M099 | 0.040791 | 1.358136 |
| N-acetylvaline | 227752 | M046 | 0.042615 | 0.483809 |
| 5,6-dihydrouracil | 649 | M148 | significant | Down |
| cadaverine | 273 | M029 | significant | UP |
| UDP-galactose | 46936243 | M077 | significant | UP |
| guanosine | 6802 | M140 | significant | Down |
| 5-methylthioadenosine (MTA) | 149 | M037 | significant | Down |
| beta-hydroxypyruvate | 964 | M024 | significant | Down |
| galactose 1-phosphate | 123912 | M062 | significant | UP |
