# Supplementary material for: Inactivation of Metabolic Genes Causes Short- and Long-Range dys-Regulation in Escherichia coli Metabolic Network
Source: PLoS One. 2013 Dec 5;8(12):e78360. doi: 10.1371/journal.pone.0078360 (PMC3868466; doi:10.1371/journal.pone.0078360)
Supplement: Table S5 — Significantly altered (Student’s T-test, p<0.05) metabolites in galU mutant strain of E. coli cultured in galactose supplemented media. (DOCX) [file pone.0078360.s007.docx]

Table S5. Significantly altered (Student TTest, p<0.05) metabolites in *galU* strain of *E. Coli* cultured in galactose supplemented media.

| **Metabolite Name** | **PubChem ID** | **Label in**  **Fig 3** | **galU p-value** | **galU fold change** |
| --- | --- | --- | --- | --- |
| N-acetylaspartate (NAA) | 65065 | M005 | 1.96E-06 | 0.22 |
| 2-aminobutyrate | 80283 | M007 | 6.84E-05 | 0.31 |
| ribose | 5779 | M075 | 0.000103 | 7.82 |
| riboflavin (Vitamin B2) | 6759 | M094 | 0.000188 | 3.98 |
| nicotinate ribonucleoside* | 121991 | M083 | 0.000254 | 0.30 |
| orotate | 967 | M142 | 0.000273 | 0.21 |
| galactose | 439357 | M053 | 0.000277 | 7677.41 |
| glutathione, reduced (GSH) | 124886 | M016 | 0.000377 | 0.08 |
| 5,6-dihydrouracil | 649 | M148 | 0.000623 | 0.11 |
| N-acetylvaline | 227752 | M046 | 0.000787 | 0.43 |
| acetyl CoA | 444493 | M090 | 0.001412 | 0.43 |
| glycine | 750 | M020 | 0.00161 | 0.48 |
| nicotinate | 938 | M082 | 0.001644 | 1.74 |
| 3-hydroxybutyrate (BHBA) | 441 | M109 | 0.001658 | 0.35 |
| pantothenate | 6613 | M088 | 0.00174 | 0.57 |
| cysteinylglycine | 439498 | M152 | 0.002355 | 3.79 |
| inosine | 6021 | M135 | 0.002655 | 0.58 |
| fructose-6-phosphate | 69507 | M072 | 0.003269 | 4.09 |
| 2-pyrrolidinone | 12025 | M155 | 0.00383 | 0.34 |
| adenosine | 60961 | M138 | 0.004097 | 0.37 |
| glutamate | 23327 | M011 | 0.004229 | 0.09 |
| 3-(4-hydroxyphenyl)lactate | 9378 | M033 | 0.004593 | 1.73 |
| cytidine 5'-monophosphate (5'-CMP) | 6131 | M141 | 0.004648 | 2.00 |
| 3-phosphoglycerate | 724 | M070 | 0.005101 | 0.20 |
| citrate | 311 | M100 | 0.005812 | 0.49 |
| homoserine | 12647 | M023 | 0.00623 | 0.10 |
| nicotinate adenine dinucleotide (NAAD+) | 165490 | M086 | 0.007398 | 0.13 |
| threonine | 6288 | M022 | 0.008857 | 0.75 |
| succinate | 1110 | M097 | 0.009078 | 0.51 |
| gamma-aminobutyrate (GABA) | 119 | M012 | 0.010243 | 0.28 |
| prolylleucine | 173815 | M151 | 0.010343 | 0.33 |
| pseudouridine | 15047 | M147 | 0.011323 | 0.69 |
| adenine | 190 | M136 | 0.011416 | 0.46 |
| diaminopimelate | 865 | M028 | 0.011858 | 0.32 |
| myristoleate (14:1n5) | 5281119 | M115 | 0.012831 | 1.76 |
| valine | 1182 | M044 | 0.013362 | 0.79 |
| alanylalanine | 601 | M150 | 0.015402 | 0.78 |
| 2-oleoylglycerophosphoethanolamine* | 9547071 | M120 | 0.015835 | 2.08 |
| malate | 525 | M099 | 0.016552 | 1.76 |
| 1-oleoylglycerol (1-monoolein) | 5283468 | M131 | 0.019199 | 1.37 |
| phenyllactate (PLA) | 643327 | M032 | 0.019642 | 2.05 |
| adenosine 5'-monophosphate (AMP) | 6083 | M137 | 0.020364 | 0.72 |
| 1-palmitoylglycerophosphoethanolamine | 89229 | M121 | 0.020824 | 3.17 |
| nicotinamide adenine dinucleotide (NAD+) | 5892 | M079 | 0.022052 | 0.41 |
| nicotinamide adenine dinucleotide reduced (NADH) | 439153 | M084 | 0.02484 | 0.68 |
| N-acetylglutamate | 185 | M013 | 0.02489 | 3.44 |
| 4-hydroxybutyrate (GHB) | 10413 | M105 | 0.025936 | 0.72 |
| agmatine | 199 | M036 | 0.029322 | 0.61 |
| nicotinic acid mononucleotide (NaMN) | 121991 | M080 | 0.030204 | 0.23 |
| maltose | 439186 | M055 | 0.031491 | 1.39 |
| glutamate, gamma-methyl ester | 68662 | M014 | 0.031527 | 0.22 |
| phosphopantetheine | 987 | M091 | 0.033532 | 2.89 |
| palmitoleate (16:1n7) | 5282745 | M112 | 0.033726 | 2.53 |
| thymine | 1135 | M144 | 0.040986 | 1.45 |
| flavin mononucleotide (FMN) | 710 | M096 | 0.044744 | 1.20 |
| histidine | 6274 | M025 | 0.048327 | 0.67 |
| ophthalmate | 193304 | M017 | Significant | Down |
| glutathione, oxidized (GSSG) | 975 | M019 | Significant | Down |
| cysteine-glutathione disulfide | 3080690 | M018 | Significant | Down |
| 2-oleoylglycerophosphocholine* | 5280634 | M125 | Significant | Up |
| 1-oleoylglycerophosphocholine | 6449974 | M123 | Significant | Up |
| Isobar: fructose 1,6-diphosphate, glucose 1,6-diphosphate | 718 | M073 | Significant | Up |
| fructose | 439709 | M054 | Significant | Down |
| succinyl CoA | 439161 | M093 | Significant | Down |
| thymidine 5'-monophosphate | 9700 | M145 | Significant | Down |
| 5-methylthioadenosine (MTA) | 149 | M037 | Significant | Down |
| galactose 1-phosphate | 123912 | M062 | Significant | Up |
| 3-deoxyoctulosonate | 5460395 | M154 | Significant | Up |
